# Genetic and clinical investigation of insulin-degrading enzyme in Parkinson’s disease within the Chinese Han population

**DOI:** 10.3389/fnins.2026.1827842

**Published:** 2026-05-18

**Authors:** Huimin Zheng, Yu Guo, Lue Zhou, Yun Su, Xin Cui, Shuyu Zhang, Zhengwei Hu, Shuangjie Li, Changhe Shi, Yuming Xu, Chengyuan Mao, Yonggang Wang

**Affiliations:** 1 Department of Neurology, The First Affiliated Hospital of Zhengzhou University, Zhengzhou University, Zhengzhou, Henan, China; 2 The Academy of Medical Sciences of Zhengzhou University, Zhengzhou University, Zhengzhou, Henan, China; 3 Henan Key Laboratory of Cerebrovascular Diseases, The First Affiliated Hospital of Zhengzhou University, Zhengzhou University, Zhengzhou, Henan, China; 4 NHC Key Laboratory of Prevention and Treatment of Cerebrovascular Diseases, Zhengzhou, Henan, China; 5 Tianjian Laboratory of Advanced Biomedical Sciences, School of Life Sciences, Zhengzhou University, Zhengzhou, Henan, China; 6 Department of Neurosurgery, The First Affiliated Hospital of Zhengzhou University, Zhengzhou University, Zhengzhou, Henan, China; 7 Department of Neurology, Beijing Tiantan Hospital, Headache Center, Capital Medical University, Beijing, China

**Keywords:** Chinese Han population, insulin-degrading enzyme, Mini-Mental State Examination, Parkinson’s disease, Single nucleotide polymorphisms

## Abstract

**Introduction:**

Growing evidence suggests a mechanistic link between type 2 diabetes mellitus and Parkinson’s disease (PD), with insulin-degrading enzyme (IDE) implicated in both insulin and amyloid-β metabolism, as well as α-synuclein degradation. However, the role of IDE in PD pathogenesis remains insufficiently defined. This study aimed to investigate the association of *IDE* gene polymorphisms and serum IDE levels with sporadic PD in a Chinese Han population.

**Methods:**

Fourteen single nucleotide polymorphisms (SNPs) within the *IDE* gene were genotyped in 463 patients with sporadic PD and 576 age- and sex-matched healthy controls (HCs). An independent cohort of 100 PD patients and 100 HCs was used to quantify serum IDE concentrations. Correlations between IDE levels and clinical features were assessed. Logistic regression was employed to identify independent factors associated with PD.

**Results:**

Among the examined SNPs, rs11187007 showed a nominal allelic association with PD (*P* = 0.046), which did not survive the Bonferroni correction. Serum IDE concentrations were significantly higher in PD patients than in HCs (*P* = 0.015). Elevated IDE levels were negatively correlated with Mini-Mental State Examination scores (*R* = –0.230, *P* = 0.027) and positively associated with more severe symptoms. Logistic regression indicated that elevated serum IDE levels were associated with PD.

**Conclusion:**

Our findings highlight that elevated serum IDE correlates with PD, suggesting a role for IDE in neurodegeneration, warranting further mechanistic and longitudinal studies to evaluate its potential as a therapeutic target in PD.

## 1 Introduction

Parkinson’s disease (PD) is the second most common neurodegenerative disorder, clinically characterized by a progressive loss of dopaminergic neurons in the substantia nigra, intracellular aggregation of α-synuclein (α-syn), and a broad spectrum of motor and non-motor symptoms (Cheong et al., 2020; Su et al., 2025). Increasing evidence suggests a significant pathological and epidemiological overlap between PD and type 2 diabetes mellitus (T2DM)—a metabolic disorder defined by insulin resistance and accumulation of islet amyloid polypeptide (IAPP) in pancreatic β-cells (Cheong et al., 2020; Meng et al., 2020). Epidemiological studies have shown that T2DM is associated with an increased risk of developing PD (Aviles-Olmos et al., 2013; Sabari et al., 2023), as well as with accelerated disease progression, worse cognitive performance, and greater motor symptom severity (Athauda et al., 2022; Sabari et al., 2023). Furthermore, impaired glucose metabolism has been observed in patients with sporadic PD, and antidiabetic agents—particularly glucagon-like peptide-1 receptor agonists—have shown neuroprotective potential in clinical trials for PD (Reich and Hölscher, 2022; Vijiaratnam et al., 2025). In addition, shared dysregulated molecular pathways, including oxidative stress, mitochondrial dysfunction, and impaired insulin receptor and lipid signaling, may reflect the interplay between metabolic dysfunction and neurodegeneration in PD and T2DM (Santiago and Potashkin, 2013a,b). Specifically, APP can serve as a biomarker to distinguish PD patients, and increased expression of APP in blood can modulate the neurodegenerative phenotype in T2DM patients (Santiago and Potashkin, 2013a). These findings highlight a potential convergence of metabolic dysregulation and protein misfolding in the pathogenesis of both T2DM and PD.

Insulin-degrading enzyme (IDE), a zinc-dependent metallopeptidase, is centrally involved in the degradation of several amyloidogenic peptides, including insulin, IAPP, amyloid-β (Aβ), and α-syn (Sousa et al., 2021; Tian et al., 2023). Structurally, IDE comprises four homologous domains, organized into N-terminal domains 1 and 2 and C-terminal domains 3 and 4, which coordinate substrate recognition and catalytic activity (Tundo et al., 2017; Sousa et al., 2021). Beyond its classical proteolytic functions, IDE also exerts non-proteolytic roles, such as chaperone-like activity and regulation of protein homeostasis via the ubiquitin–proteasome system, thus implicating IDE in the pathophysiology of neurodegenerative diseases (Sousa et al., 2021). Genome-wide association studies have linked *IDE* polymorphisms to susceptibility for T2DM and Alzheimer’s disease (AD) (Mueller et al., 2007; Zhou et al., 2010; Kim et al., 2018), and altered serum IDE levels have been reported in both disorders (Kullenberg et al., 2022, 2023). In the context of PD, IDE has been shown to interact with α-syn, potentially modulating its aggregation and deposition (Sharma et al., 2015a,b). Recently, a mechanistic study supported IDE’s neuroprotective role in PD (Zheng et al., 2025). However, the key role of IDE in PD remains poorly understood, and the respective relationships of *IDE* genetic polymorphisms and serum IDE levels with sporadic PD remain to be elucidated.

In this study, we investigated the genetic and biochemical characteristics of IDE in a Han Chinese cohort with sporadic PD. Specifically, we genotyped 14 single nucleotide polymorphisms (SNPs) within the *IDE* gene and quantified serum IDE concentrations. We also assessed whether serum IDE levels were associated with disease severity, including MDS-UPDRS scores and cognitive performance measured by the Mini-Mental State Examination (MMSE). Finally, logistic regression analysis was performed to evaluate the association between serum IDE levels and PD.

## 2 Materials and methods

### 2.1 Participants and clinical assessment

We enrolled 463 sporadic PD patients and 576 matched healthy controls (HCs) for genetic analyses and a separate cohort of 100 PD patients and 100 HCs for biochemical tests. All participants were recruited from The First Affiliated Hospital of Zhengzhou University (2012–2022). All PD patients were assessed using the Movement Disorders Society clinical diagnostic criteria (Postuma et al., 2015). Individuals with malignancies, autoimmune diseases, T2DM, cardiovascular diseases, or other neurodegenerative disorders were excluded.

**FIGURE 1:**
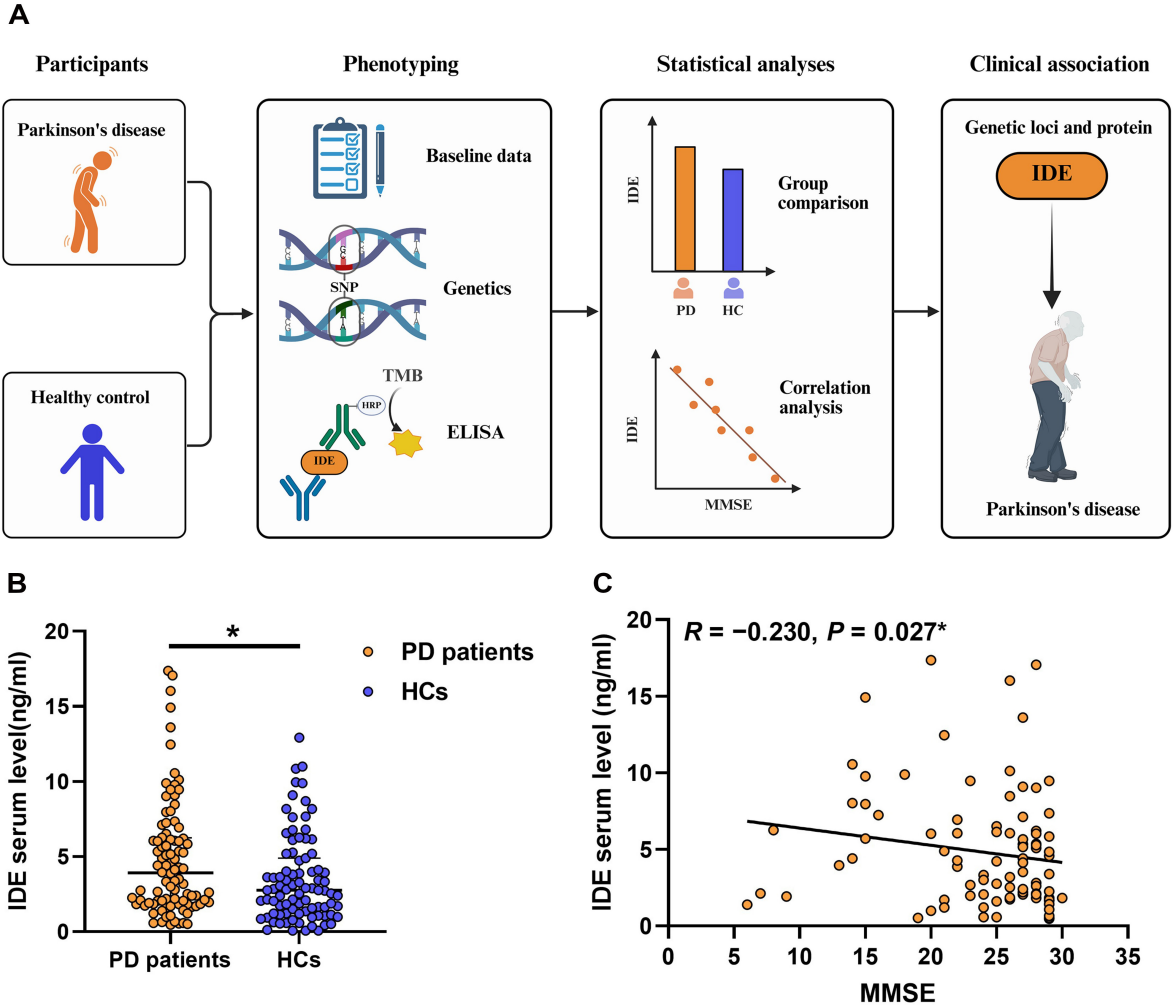
Elevated serum IDE levels and their association with cognitive decline in Parkinson’s disease. **(A)** Workflow for experiments in blood samples from PD patients and healthy controls. Elements were partially created with BioRender.com. **(B)** Higher serum IDE levels were observed in PD patients compared with HCs. *P < 0.05, Mann-Whitney U test. **(C)** Bivariate association analysis revealed that increased serum IDE levels were associated with poorer MMSE scores. * P < 0.05, Spearman’s correlation test. PD, Parkinson’s disease; HCs, healthy controls; MMSE, Mini-Mental State Examination; IDE, insulin-degrading enzyme.

The graphical workflow of the study design was illustrated in Figure 1A. All PD patients were evaluated in the morning in the “off” state, without receiving any medication. The severity and clinical phenotype of PD were determined using the modified Hoehn and Yahr staging scale and specific Movement Disorder Society-Unified Parkinson’s Disease Rating (MDS-UPDRS) items (Goetz et al., 2008). Clinical data in PD patients included demographics (age, sex), disease duration, age at onset (AAO), Hoehn and Yahr stage, MDS-UPDRS scores, and levodopa-equivalent daily doses (LEDDs) (Schade et al., 2020). Cognitive function was assessed by the MMSE, with cut-off values adjusted for years of education. Additionally, metabolic indicators such as fasting blood glucose (FBG), hemoglobin A1c (HbA1c), body mass index (BMI), and weight were evaluated for all subjects. This study was approved by the Institutional Research Ethics Committee; all participants or their guardians provided written informed consent.

### 2.2 DNA extraction and SNP genotyping

Peripheral blood samples were collected after an overnight fast. Genomic DNA was isolated using a standard kit protocol (BioTeke, Beijing, China). Fourteen selected *IDE* SNPs were genotyped by improved multiple ligase detection reaction (iMLDR). The selection of SNPs was based on previous studies linking *IDE* polymorphisms to AD and T2DM, which covers the *IDE* gene region on chromosome 10 (Sakai et al., 2004; Blomqvist et al., 2005; Liang et al., 2007; Mueller et al., 2007; Rudovich et al., 2009; Carrasquillo et al., 2010; Zou et al., 2010; Wang et al., 2012). Furthermore, we searched the relevant *IDE* SNPs in the NCBI dbSNP and the Ensembl Genome websites, and performed linkage disequilibrium (LD) analyses to reduce redundancy.

### 2.3 Serum IDE quantification

Serum was separated by centrifugation (4°C, 10 min, 1,000 g) and stored at −80°C. IDE levels were measured by enzyme-linked immunosorbent assay (Cloud-Clone, Houston, TX, United States). After incubating serum samples with specific IDE antibodies, horseradish peroxidase-conjugated secondary antibodies, and tetramethylbenzidine substrate, optical density was read at 450 nm. IDE concentrations were determined via a standard curve and expressed in ng/mL. The assay’s minimum detectable dose for IDE was 0.59 ng/mL. Of note, PD patients were divided into high-IDE and low-IDE groups based on the median serum IDE levels in subgroup analyses.

### 2.4 Statistical analyses

Prior to analysis, outliers (determinates exceeding ± 3 standard deviations from the mean) were removed. Continuous variables were presented as mean ± standard deviation (SD) or median with interquartile range, depending on their distribution. Hardy-Weinberg equilibrium tests and minor allele frequency calculations were performed for each SNP. For categorical variables, Chi-square tests or Fisher’s exact tests evaluated the distribution of alleles/genotypes, yielding *P*-values, odds ratios (OR), and 95% confidence intervals (CI). A Bonferroni correction was applied for multiple testing to determine statistical significance.

Continuous variables were tested for normal distribution with the Shapiro-Wilk test. Comparisons between two groups were analyzed using the Mann-Whitney U test or Student’s *t*-test, depending on the data distribution. Multiple comparisons with more than two groups data were conducted by either one-way ANOVA (followed by pairwise comparisons with *post-hoc* Tukey tests) or Kruskal-Wallis test (followed by pairwise comparisons with *post-hoc* Dunn’s test), depending on the data distribution. Spearman’s rank correlation assessed relationships between serum IDE levels and clinical metrics. Logistic regression analysis determined the association between serum IDE levels and PD, and age, gender, FBG, HbA1c, BMI, and weight factors were adjusted in multivariate regression analysis. Statistical significance was accepted at *P* < 0.05 (two-tailed). All statistical analyses were performed with IBM SPSS software (version 29.0), and plots were produced using GraphPad Prism (version 9.0.0).

## 3 Results

### 3.1 Participant characteristics

A total of 463 patients with PD and 576 HCs were included in the genetic analysis. Serum IDE levels were measured in an independent subset comprising 100 PD patients and 100 HCs (Table 1). The PD group had a mean age of 59.45 ± 8.58 years, with an average disease duration of 4.44 ± 3.17 years and a mean AAO of 54.76 ± 9.03 years. The average MDS-UPDRS Part III score was 39.31 ± 19.16, the mean LEDDs was 381.70 ± 339.99 mg, and the mean Hoehn and Yahr stage was 2.10 ± 0.85. No significant differences were observed between PD patients and HCs in terms of FBG, HbA1c, BMI, or weight (Table 1).

**TABLE 1:** Demographic and clinical characteristics of Parkinson’s disease patients and HCs, and serum IDE levels increased in PD patients.

Variable	PD patients (*n* = 100)	HCs (*n* = 100)	*p*
Gender, n (female/male)	47/53	50/50	0.671
Age, y, mean ± SD	59.45 ± 8.58 8.58246 ± 9.75	57.44 ± 7.36	0.079
AAO, y, mean ± SD	54.76 ± 9.03	NA	NA
Duration of PD, y, mean ± SD (range)	4.44 ± 3.17 (1–17)	NA	NA
MDS-UPDRS I, mean ± SD (range)	8.18 ± 5.73 (0–27)	NA	NA
MDS-UPDRS II, mean ± SD (range)	13.80 ± 7.77 (1–42)	NA	NA
MDS-UPDRS III, mean ± SD (range)	39.31 ± 19.16 (7–116)	NA	NA
LEDDs, mg, mean ± SD (range)	381.70 ± 339.99 (0–2060)	NA	NA
Hoehn and Yahr stage, mean ± SD (range)	2.10 ± 0.85 (1–5)	NA	NA
MMSE, mean ± SD (range)	24.30 ± 5.50 (6–30)	NA	NA
FBG, mmol/L, mean ± SDl	4.70 ± 0.51	4.71 ± 0.56	0.592
HbA1c, mmol/L, mean ± SD	5.53 ± 0.39	5.63 ± 0.36	0.114
BMI, kg/m^2^, mean ± SD	23.06 ± 3.16	23.59 ± 3.09	0.256
Weight, kg, mean ± SD	63.49 ± 10.55	63.35 ± 10.78	0.689
IDE, ng/mL, median (QL–QU)	3.92 (1.87– 6.25)	2.76 (1.21–4.89)	0.015*

Elevated IDE serum levels in PD patients reached statistical significance compared to HCs. Categorical variables were compared by using the Pearson chi-square test. Continuous variables in normal distribution and homogeneity of variance were compared by using the two-sample *t*-test; otherwise, the Mann-Whitney U test were performed.

*P < 0.05, Mann-Whitney U test. PD, Parkinson’s disease; HCs, healthy controls; AAO, age at PD onset; MDS-UPDRS, Movement Disorder Society-Unified Parkinson’s Disease Rating Scale; LEDDs, levodopa-equivalent daily doses; MMSE, Mini-Mental State Examination; FBG, fasting blood glucose; HbA1c, hemoglobin A1c; BMI, body mass index; IDE, insulin-degrading enzyme; NA, not available; n, number.

### 3.2 Association between IDE gene polymorphisms and PD

Fourteen *IDE* SNPs previously associated with T2DM or AD were selected for genotyping using the improved iMLDR method (Sakai et al., 2004; Blomqvist et al., 2005; Liang et al., 2007; Mueller et al., 2007; Rudovich et al., 2009; Carrasquillo et al., 2010; Zou et al., 2010; Wang et al., 2012). These SNPs were identified through literature review and database searches (NCBI dbSNP and Ensembl), followed by LD analyses to minimize redundancy. The chromosomal positions, rsIDs, and location annotations of these variants are provided in Supplementary Table S1. Notably, all SNPs were located in non-coding regions of the *IDE* gene, including introns, 5’ flanking, and 3’ flanking regions. All 14 SNPs conformed to Hardy–Weinberg equilibrium in both PD and HC groups (*P* > 0.05). The minor allele frequencies (MAFs) of rs12783634, rs17875327, and rs4646955 were below 0.05 (Supplementary Table S2). Among the analyzed SNPs, rs11187007 showed a nominal allelic association with PD in the crude analysis (*P* = 0.046, OR = 1.217, 95% CI = 1.003–1.476, Table 2). However, this significance did not survive after Bonferroni correction for multiple testing (Bonferroni-corrected *P* = 0.644). No significant associations were found under the recessive or dominant genetic models, and the remaining 13 SNPs showed no significant associations with PD (Supplementary Table S2).

**TABLE 2:** Assessment of the relationship level of *IDE* SNPs with PD in the Chinese Han population.

SNP	HWE (*p-*value)	MAF	Association test	PD patients	HCs	*P* ^#^	OR (95%CI)
rs11187007	1.000	0.724	Genotypic (GG/GA/AA)	228/194/41	316/222/38	–	–
			Dominant (GG+GA/AA)	422/41	538/38	0.172	1.376 (0.869–2.178)
			Recessive (GG/GA+AA)	228/235	316/260	0.072	1.253 (0.980–1.601)
			Alleles (G/A)	650/276	854/298	**0.046(0.644)**	**1.217 (1.003–1.476)**

The SNPs of the *IDE* gene located on Chromosome 10. The *P-*value of the Hardy-Weinberg equilibrium test and the minor allele frequency (MAF) were calculated for each SNP. The Chi-square test and Fisher’s exact test displayed the distribution of the association tests with *p*-values, odds ratios (OR), and confidence intervals (95% CI) for SNPs of the *IDE* gene. ^#^ *P*-values were corrected for multiple testing using Bonferroni correction (in parentheses). Nominal significance was defined as an uncorrected *P* < 0.05, and statistical significance was defined as a Bonferroni-corrected *P* < 0.05. Bold values indicate the nominal statistical significance. IDE, insulin-degrading enzyme; PD, Parkinson’s disease; HCs, healthy controls; SNPs, single nucleotide polymorphisms; HWE, Hardy-Weinberg equilibrium; MAF, minor allele frequency; CI, confidence interval; OR, odds ratio.

### 3.3 Elevated serum IDE levels in PD and their clinical correlates

Serum IDE levels were significantly elevated in PD patients compared to healthy controls (3.92 vs. 2.76 ng/mL, *P* = 0.015; Figure 1B; Table 1). To further explore the clinical significance of IDE elevation, PD patients were stratified into high- and low-IDE subgroups based on the median IDE level. Patients in the high-IDE subgroup exhibited higher MDS-UPDRS Part I scores and greater LEDD values than those in the low-IDE subgroup (Table 3).

**TABLE 3:** Comparison between high and low serum IDE level groups in patients with PD.

Variable	Low IDE (*n* = 50)	High IDE (*n* = 50)	*p*
Gender, n (female/male)	24/26	23/27	0.841
Age, y, mean ± SD	59.00 ± 9.27	59.83 ± 8.31	0.654
AAO, y, mean ± SD	54.78 ± 9.35	55.02 ± 8.69	0.997
Duration of PD, y, mean ± SD (range)	4.26 ± 2.76(1–13)	4.80 ± 3.67(1–17)	0.717
MDS-UPDRS I, mean ± SD (range)	6.96 ± 4.99(0–22)	9.67 ± 6.37(1–27)	0.034*
MDS-UPDRS II, mean ± SD (range)	12.50 ± 6.87(1–31)	15.22 ± 8.45(6–42)	0.101
MDS-UPDRS III, mean ± SD (range)	35.43 ± 17.14(8–70)	43.74 ± 21.03(7–116)	0.081
LEDDs, mg, mean ± SD (range)	265.11 ± 237.32(0–950)	472.72 ± 388.55(0–2060)	0.003**
Hoehn and Yahr stage, mean ± SD (range)	1.84 ± 0.79(1–4)	2.19 ± 0.97(1–5)	0.083
MMSE, mean ± SD (range)	25.02 ± 5.48(6–30)	23.13 ± 5.69(8–29)	0.050

Based on the median value of IDE, PD patients were divided into the high-IDE and low-IDE groups. There was no statistically significant difference in age and gender between the two groups. Positive results were found that the high-IDE group had higher MDS-UPDRS I scores and LEDDs than the low-IDE group. Categorical variables were compared by using the Pearson chi-square test. Continuous variables in normal distribution and homogeneity of variance were compared by using the two-sample *t*-test; otherwise, the Mann-Whitney U test were performed.

*P < 0.05,

**P < 0.01, Mann-Whitney U test. PD, Parkinson’s disease; HCs, healthy controls; AAO, age at PD onset; MDS-UPDRS, Movement Disorder Society-Unified Parkinson’s Disease Rating Scale; LEDDs, levodopa-equivalent daily doses; MMSE, Mini-Mental State Examination; IDE, insulin-degrading enzyme; n, number.

Additional subgroup analyses revealed no significant associations between serum IDE and clinical or metabolic parameters, including sex, disease duration, AAO, or Hoehn and Yahr stage (Supplementary Tables S3–S8). Pearson correlation analysis revealed a weak but statistically significant negative correlation between serum IDE levels and MMSE scores (*R* = –0.230, *P* = 0.027; Figure 1C), suggesting a potential link between IDE and cognitive impairment. No significant correlations were observed between IDE levels and other clinical features or metabolic markers (Supplementary Table S9).

Finally, both univariate and multivariate logistic regression analyses confirmed that elevated serum IDE levels were associated with PD (Table 4), independent of age, sex, and metabolic variables.

**TABLE 4:** Logistic regressive analyses.

Variable	OR (95%CI)	*p*
IDE, ng/mL		
Univariate analysis	1.127 (1.027–1.238)	0.012*
Multivariate analysis^a^	1.156 (1.044–1.281)	0.005**

Univariate and multivariate logistic regression analyses showed a significant correlation between elevated serum IDE levels and PD.

*P < 0.05,

**P < 0.01, Logistic regressive analyses.

^a^ multivariate regression analysis after adjusting for age, gender, FBG, HbA1c, BMI, and weight factors. IDE, insulin-degrading enzyme; OR, odds ratio; 95% CI, 95% confidence interval.

## 4 Discussion

In this study, we investigated the genetic variants and serum protein levels of IDE in a Han Chinese cohort with sporadic PD. We identified a nominal allelic association between the *IDE* rs11187007 SNP and PD, although it lost statistical significance after applying Bonferroni corrections. Moreover, serum IDE levels were markedly elevated in PD patients compared to healthy controls, with higher IDE levels correlating with more severe symptoms and lower MMSE scores. These findings suggest that IDE dysregulation may contribute to PD pathogenesis and disease severity.

IDE is located on chromosome 10q23–q25 and has been genetically linked to both T2DM and AD (Mueller et al., 2007; Zhou et al., 2010; Wang et al., 2012). Previous studies have identified that *IDE* SNPs rs11187007 (Chinese populations) and rs1887922 (German populations) have been linked to T2DM risk (Rudovich et al., 2009; Zhou et al., 2010). Similarly, *IDE* risk variants were found in AD, including rs4646955 (Finnish cohorts) (Vepsäläinen et al., 2007), rs11187007 (German cohorts) (Mueller et al., 2007), and rs3781239 (Chinese population) (Wang et al., 2012). Several *IDE* variants have also been correlated with Aβ levels in plasma and brain tissue, and cognitive performance (Blomqvist et al., 2005; Reitz et al., 2012). Interestingly, subgroup analyses indicated an age-modified effect for rs12783634 and a sex-independent effect for rs1887922 in AD (Blomqvist et al., 2005; Mueller et al., 2007). These variations in *IDE* polymorphism reflect differences in distinct population-specific effects and gene-environment interactions in T2DM and AD patients. Interestingly, in an Australian cohort, *IDE* variants were associated with early-onset PD (Blomqvist et al., 2004). In our study, the rs11187007 variant—previously implicated in T2DM and AD (Mueller et al., 2007; Zhou et al., 2010)—was nominally associated with PD, raising the possibility of shared pathogenic mechanisms (e.g., altered insulin signaling, IDE enzymatic activity, and amyloid clearance) involving IDE among these diseases. The nominal significance failed to survive after the stringent Bonferroni correction, which can be attributed to the limited sample size, warranting larger clinical cohort in the future. Given that rs11187007 is in a non-coding region, it likely affects gene regulation rather than the IDE protein structure or enzymatic function. Interestingly, the deCODE database reports that the effect allele of rs11187007 is linked to increased plasma IDE levels (Eldjarn et al., 2023) aligns with our observation of elevated serum IDE in PD patients, further suggesting an association between IDE and PD.

IDE is ubiquitously expressed in both intracellular and extracellular compartments and can be measured in serum (Leissring et al., 2004; Bulloj et al., 2008; Bulloj et al., 2010; Kullenberg et al., 2022). Prior studies have reported reduced brain IDE levels in diabetic models (Kochkina et al., 2015), while higher serum IDE levels were found in T2DM compared to both AD patients and healthy controls (Kullenberg et al., 2022, 2024). Conversely, IDE levels and activity were reported to be decreased in AD, potentially reflecting disease-specific regulation and inflammation-related impairment (Liu et al., 2012; da Costa et al., 2017; Kullenberg et al., 2023). A recent mechanistic study demonstrated that IDE overexpression alleviated motor deficits and reduced α-syn pathology in A53T α-syn mice, indicating a neuroprotective role of IDE in PD (Zheng et al., 2025). Our findings extend the literature by showing increased serum IDE in PD, adding complexity to the interpretation of IDE dysregulation in neurodegenerative diseases, which can be attributed to differences between central and peripheral IDE measurements. Notably, our results suggested that elevated serum IDE levels independently correlated with the severity of PD, including worse clinical and cognitive scores.

A proposed model posits that increased IDE may reflect a compensatory response to reduced enzymatic activity due to chronic inflammation or insulin resistance (Kullenberg et al., 2022, 2024). Consistently, IDE-deficient animal models exhibit α-syn accumulation and aggregation (Steneberg et al., 2013; Merino et al., 2022). We hypothesize that elevated IDE may reflect dysregulated insulin signaling, altered enzymatic activity, and α-syn pathology. However, direct measurements of IDE enzymatic activity and its interactions with α-syn are required to substantiate this hypothesis.

IDE’s role in synaptic function and cognitive integrity is increasingly recognized. It maintains insulin pathway homeostasis and modulates synaptic plasticity, potentially affecting cognition (Chiu et al., 2008; Tian et al., 2023). The weak, albeit statistically significant, negative correlation observed between serum IDE levels and MMSE scores suggests a potential link between IDE dysregulation and cognitive decline in PD, possibly mediated by impaired insulin signaling. However, these preliminary observations warrant further investigation in larger longitudinal cohorts, coupled with mechanistic studies.

Although the precise molecular mechanisms linking IDE to PD remain elusive, three interrelated pathways may be involved: 1. Direct interaction with α-syn: IDE may bind to the C-terminal domain of α-syn and prevent its aggregation through a non-proteolytic mechanism (Sharma et al., 2015a,b). Dysfunctional IDE could impair this protective interaction, facilitating the formation of toxic α-syn fibrils (Merino et al., 2022). 2. Proteostasis disruption: The “dead-end chaperone” hypothesis proposes that aberrant IDE impairs α-syn degradation via the autophagy-lysosome and proteasome systems, contributing to protein accumulation and neuronal toxicity (de Tullio et al., 2008; Sousa et al., 2021). 3. Insulin signaling impairment: Chronic insulin resistance and IDE dysregulation may exacerbate dopaminergic neuronal vulnerability, hinder synaptic integrity, and promote PD progression (Kleinridders et al., 2015).

Our study provides evidence that increased serum IDE in PD may reflect a pathological or compensatory process. However, proteomic analyses in PD have reported no consistent differences in IDE levels across the substantia nigra, cerebrospinal fluid (CSF), plasma, and urine (Jang et al., 2023; Rutledge et al., 2024). These discrepancies likely stem from variations in sample sources, disease stages, technical platforms, and study populations. Future studies should integrate IDE quantification across serum, CSF, and brain tissue, along with functional *in vitro* assays, to clarify whether IDE facilitates or mitigates α-syn pathology.

Several limitations warrant discussion: 1. Exploratory nature and sample size: Exploratory candidate-SNP approach and moderate cohort size constrained the statistical power to detect SNP associations. In addition, the weak but statistically significant correlations between serum IDE levels and clinical measures should be cautiously interpreted with limited sample size, warranting more validation and evidence to establish serum IDE as a biomarker in larger longitudinal cohorts. 2. Lack of brain tissue IDE measurements: We did not assess IDE expression or enzymatic activity in the brain, which precludes direct evaluation of the functional impact of the rs11187007 variant. Although serum IDE may reflect systemic alterations, brain-specific IDE assays are necessary to confirm our findings. 3. Separate cohorts and cross-sectional design: Our nominal genetic findings lack validation in replication cohort and longitudinal data, limiting the generalizability and temporal causality. The absence of longitudinal data precludes assessing IDE during PD progression, necessitating prospective studies to longitudinally measure serum and brain IDE levels. Genetic and serum analyses were conducted in two separate cohorts, which precluded the evaluation of the interactive influence of *IDE* genotypes and serum IDE levels. Paired genetic and serum datasets from the same cohort are necessary in the future. 4. Undetermined metabolic status and insulin resistance: Despite excluding T2DM patients and adjusting for metabolic variables (FBG, HbA1c, BMI, and weight), unmeasured fasting insulin levels and HOMA-IR index could still exert confounding effects on IDE expression via insulin resistance—a possibility that requires consideration and mechanistic research in future studies.

Consequently, future research should expand cohort sizes, incorporate independent replication and longitudinal follow-up, and conduct metabolic, enzymatic, and mechanistic investigations using human, cellular, and animal models to elucidate IDE’s role in PD pathogenesis.

## 5 Conclusion

In summary, our study identified that elevated serum IDE levels independently correlated with the severity of Parkinson’s disease. Furthermore, we identified a nominal allelic association between the *IDE* rs11187007 SNP and PD, suggesting a potential genetic contribution that warrants further validation in larger cohorts. These findings raise the possibility that IDE is associated with PD, potentially involving altered insulin signaling, IDE enzymatic activity, and α-syn clearance. Further investigations using larger, multi-center cohorts and longitudinal designs are needed to validate these associations. In addition, mechanistic studies exploring IDE’s enzymatic activity, regulatory pathways, and interaction with α-syn may offer valuable insights into its therapeutic potential. Ultimately, targeting IDE may represent a promising avenue for developing novel disease-modifying strategies in PD.

## Data Availability

The original contributions presented in the study are included in the article/Supplementary material, further inquiries can be directed to the corresponding author.
