# Supplementary material for: Genetic and clinical investigation of insulin-degrading enzyme in Parkinson’s disease within the Chinese Han population
Source: Front Neurosci. 2026 May 18;20:1827842. doi: 10.3389/fnins.2026.1827842 (PMC13222982; doi:10.3389/fnins.2026.1827842)
Supplement: Supplementary file 1 [file Data_Sheet_1.docx]

Supplementary Material

**Table S1.** Specific location information for 14 *IDE* SNPs.

| **SNP (rsID)** | **Position (CHR:BP)** | **Location region** |
| --- | --- | --- |
| rs11187007 | 10:92454823 | Intron 24 |
| rs11187060 | 10:92544375 | Intron 1 |
| rs12783634 | 10:92573485 | Intron 1 |
| rs17875327 | 10:92515052 | Intron 4 |
| rs1855915 | 10:92534086 | Intron 3 |
| rs1887922 | 10:92464408 | Intron 20 |
| rs2421942 | 10:92527623 | Intron 4 |
| rs3758505 | 10:92575021 | 5’ flanking |
| rs3781239 | 10:92458040 | Intron 22 |
| rs4646955 | 10:92534534 | Intron 3 |
| rs4646957 | 10:92470155 | Intron 18 |
| rs5786996 | 10:92452778 | 3’ flanking |
| rs7099761 | 10:92576042 | 5’ flanking |
| rs7910977 | 10:92450119 | 3’ flanking |

Fourteen selected single nucleotide polymorphisms (SNPs) of the *IDE* gene were listed with the rsID. The position of the SNP was displayed with chromosome: base pair (CHR: BP) according to the GRCh38/hg38 reference genome in the NCBI dbSNP websites. The location region of the SNPs was searched in the Ensembl genome websites, which were relative to reference mRNA of IDE (ENST00000398142, also known as IDE-201).

**Abbreviation:** IDE, insulin-degrading enzyme; SNPs, single nucleotide polymorphisms; CHR, chromosome; BP, base pair.**Table S2** Evaluation of the association between *IDE* SNPs and PD in the Chinese Han population.

| **SNP** | **HWE (*p-*value)** | | | **MAF** | | **Association test** | | **PD patients** | | **HCs** | | ***p*** | | **OR (95%CI)** | |  | | |
| --- | --- | --- | --- | --- | --- | --- | --- | --- | --- | --- | --- | --- | --- | --- | --- | --- | --- | --- |
| rs11187060 | | 0.553 | 0.195 | | Genotypic (TT/TC/CC) | | 20/142/301 | | 372/197/17 | | - | | - | |  | | |  |
|  | |  |  | | Dominant (TT+TC/CC) | | 162/301 | | 208/368 | | 0.707 | | 1.050 (0.813-1.356) | |  | | |  |
|  | |  |  | | Recessive (TT/TC+CC) | | 20/443 | | 16/560 | | 0.177 | | 0.633 (0.324-1.236) | |  | |  |  |
|  | |  |  | | Alleles (T/C) | | 182/744 | | 224/928 | | 0.904 | | 0.987 (0.793-1.227) | |  | |  |  |
| rs12783634 | | 1.000 | 0.028 | | Genotypic (AA/GA/GG) | | 0/25/438 | | 0/34/542 | | - | | - | |  | |  |  |
|  | |  |  | | Dominant (AA+GA/GG) | | 25/438 | | 34/542 | | 0.728 | | 1.099 (0.646-1.870) | |  | |  |  |
|  | |  |  | | Recessive (AA/GA+GG) | | 0/463 | | 0/576 | | - | | - | |  | |  |  |
|  | |  |  | | Alleles (A/G) | | 25/901 | | 34/1118 | | 0.731 | | 1.096 (0.649-1.851) | |  | |  |  |
| rs17875327 | | 1.000 | 0.002 | | Genotypic (GG/GA/AA) | | 0/3/460 | | 0/2/574 | |  | |  | |  | |  |  |
|  | |  |  | | Dominant (GG+GA/AA) | | 3/460 | | 2/574 | | 0.806 | | 0.534 (0.089-3.211) | |  | |  |  |
|  | |  |  | | Recessive (GG/GA+AA) | | 0/463 | | 0/576 | | - | | - | |  | |  |  |
|  | |  |  | | Alleles (G/A) | | 3/923 | | 2/1150 | | 0.807 | | 0.535 (0.089-3.209) | |  | |  |  |
| rs1855915 | | 1.000 | 1.000 | | Genotypic (CC) | | 463 | | 576 | | - | | - | |  | |  |  |
|  | |  |  | | - | | - | | - | | - | | - | |  | |  |  |
|  | |  |  | | - | | - | | - | | - | | - | |  | |  |  |
|  | |  |  | | Alleles (C) | | 926 | | 1152 | | - | | - | |  | |  |  |
| rs1887922 | | 0.143 | 0.920 | | Genotypic (TT/TC/CC) | | 383/78/2 | | 492/83/1 | | - | | - | |  | |  |  |
|  | |  |  | | Dominant (TT+TC/CC) | | 461/2 | | 575/1 | | 0.849 | | 2.495 (0.225-27.597) | |  | |  |  |
|  | |  |  | | Recessive (TT/TC+CC) | | 383/80 | | 492/84 | | 0.236 | | 1.223 (0.876-1.709) | |  | |  |  |
|  | |  |  | | Alleles(T/C) | | 844/82 | | 1067/85 | | 0.218 | | 1.220 (0.889-1.674) | |  | |  |  |
| rs2421942 | | 0.762 | 0.053 | | Genotypic (GG/GC/CC) | | 1/43/419 | | 2/61/513 | | - | | - | |  | |  |  |
|  | |  |  | | Dominant (GG+GC/CC) | | 44/419 | | 63/513 | | 0.450 | | 1.169 (0.779-1.755) | |  | |  |  |
|  | |  |  | | Recessive (GG/GC+CC) | | 1/462 | | 2/574 | | 1.000 | | 1.610 (0.146-17.808) | |  | |  |  |
|  | |  |  | | Alleles (G/C) | | 45/881 | | 65/1087 | | 0.428 | | 1.171 (0.792-1.730) | |  | |  |  |
| rs3758505 | | 0.057 | 0.111 | | Genotypic (CC/CA/AA) | | 8/90/365 | | 11/102/463 | | - | | - | |  | |  |  |
|  | |  |  | | Dominant (CC+CA/AA) | | 98/365 | | 113/463 | | 0.538 | | 0.909 (0.671-1.231) | |  | |  |  |
|  | |  |  | | Recessive (CC/CA+AA) | | 8/455 | | 11/565 | | 0.828 | | 1.107 (0.442-2.776) | |  | |  |  |
|  | |  |  | | Alleles (C/A) | | 106/820 | | 124/1028 | | 0.622 | | 0.933 (0.709-1.229) | |  | |  |  |
| rs3781239 | | 0.881 | 0.118 | | Genotypic (GG/GC/CC) | | 9/93/361 | | 6/122/448 | | - | | - | |  | |  |  |
|  | |  |  | | Dominant (GG+GC/CC) | | 102/361 | | 128/448 | | 0.941 | | 1.011 (0.753-1.358) | |  | |  |  |
|  | |  |  | | Recessive (GG/GC+CC) | | 9/454 | | 6/570 | | 0.226 | | 0.531 (0.188-1.503) | |  | |  |  |
|  | |  |  | | Alleles (G/C) | | 111/815 | | 134/1018 | | 0.803 | | 0.966 (0.739-1.263) | |  | |  |  |
| rs4646955 | | 0.624 | 0.033 | | Genotypic (GG/GA/AA) | | 0/33/430 | | 0/36/540 | | - | | - | |  | |  |  |
|  | |  |  | | Dominant (GG+GA/AA) | | 33/430 | | 36/540 | | 0.572 | | 0.869 (0.533-1.417) | |  | |  |  |
|  | |  |  | | Recessive (GG/GA+AA) | | 0/463 | | 0/576 | | - | | - | |  | |  |  |
|  | |  |  | | Alleles (G/A) | | 33/893 | | 36/1116 | | 0.579 | | 0.873 (0.540-1.411) | |  | |  |  |
| rs4646957 | | 0.248 | 0.251 | | Genotypic (TT/TC/CC) | | 31/180/252 | | 27/226/323 | | - | | - | |  | |  |  |
|  | |  |  | | Dominant (TT+TC/CC) | | 211/252 | | 253/323 | | 0.595 | | 0.935 (0.731-1.196) | |  | |  |  |
|  | |  |  | | Recessive (TT/TC+CC) | | 31/432 | | 27/549 | | 0.161 | | 0.685 (0.403-1.166) | |  | |  |  |
|  | |  |  | | Alleles (T/C) | | 242/684 | | 280/872 | | 0.339 | | 0.908 (0.744-1.107) | |  | |  |  |
| rs5786996 | | 1.000 | 0.056 | | Genotypic (TGTG/TGT/TT) | | 1/45/417 | | 2/65/509 | | - | | - | |  | |  |  |
|  | |  |  | | Dominant (TGTG+TGT/TT) | | 46/417 | | 67/509 | | 0.383 | | 1.193 (0.802-1.775) | |  | |  |  |
|  | |  |  | | Recessive (TGTG/TGT+TT) | | 1/462 | | 2/574 | | 1.000 | | 1.61 (0.146-17.808) | |  | |  |  |
|  | |  |  | | Alleles (TG/T) | | 47/879 | | 69/1083 | | 0.367 | | 1.192 (0.814-1.744) | |  | |  |  |
| rs7099761 | | 0.823 | 0.704 | | Genotypic (GG/GA/AA) | | 220/197/46 | | 293/240/43 | | - | | - | |  | |  |  |
|  | |  |  | | Dominant (GG+GA/AA) | | 417/46 | | 533/43 | | 0.157 | | 1.367 (0.885-2.113) | |  | |  |  |
|  | |  |  | | Recessive (GG/GA+AA) | | 220/243 | | 293/283 | | 0.283 | | 1.144 (0.895-1.461) | |  | |  |  |
|  | |  |  | | Alleles (G/A) | | 673/289 | | 826/326 | | 0.149 | | 1.15 (0.951-1.389) | |  | |  |  |
| rs7910977 | | 0.572 | 0.165 | | Genotypic (TT/TC/CC) | | 13/125/325 | | 12/167/397 | | - | | - | |  | |  |  |
|  | |  |  | | Dominant (TT+TC/CC) | | 138/325 | | 179/397 | | 0.658 | | 1.062 (0.814-1.386) | |  | |  |  |
|  | |  |  | | Recessive (TT/TC+CC) | | 13/450 | | 12/564 | | 0.449 | | 0.736 (0.333-1.630) | |  | |  |  |
|  | |  |  | | Alleles (T/C) | | 151/775 | | 191/961 | | 0.867 | | 1.020 (0.808-1.288) | |  | |  |  |

The analyzed SNPs of the IDE gene are situated on chromosome 10. The *P-*value of the Hardy-Weinberg equilibrium test and the minor allele frequency (MAF) were calculated for each SNP. The Chi-square test and Fisher's exact test displayed the distribution of the association tests with *p*-values, odds ratios (OR), and confidence intervals (95% CI) for SNPs of the *IDE* gene. *P*-values were corrected for multiple testing using Bonferroni correction.

**Abbreviation:** IDE, insulin-degrading enzyme; PD, Parkinson’s disease; HCs, healthy controls; SNPs, single nucleotide polymorphisms; HWE, Hardy-Weinberg equilibrium; MAF, minor allele frequency; CI, confidence interval; OR, odds ratio.

**Table S3.** Serum IDE levels and clinical features according to with or without cognitive impairment in patients with PD.

| **Variable** | **PD patients with**  **cognitive impairment (n=23)** | **PD patients without**  **cognitive impairment (n=77)** | ***p*** |
| --- | --- | --- | --- |
| Gender, n (female/male) | 10/13 | 37/40 | 0.700 |
| Age, y, mean ± SD | 61.22 ± 8.31 | 58.57 ± 9.12 | 0.358 |
| AAO, y, mean ± SD | 56.04 ± 7.64 | 54.38 ± 9.42 | 0.517 |
| Duration of PD, y, mean ± SD (range) | 5.22 ± 4.09 (1–15) | 4.21 ± 2.83 (1–17) | 0.533 |
| LEDDs, mg, mean ± SD (range) | 394.13 ± 310.52 (0–950) | 377.99 ± 350.15 (0–2060) | 0.705 |
| Hoehn and Yahr stage, mean ± SD (range) | 2.33 ± 1.02 (1–5) | 2.03 ± 0.79 (1–5) | 0.261 |
| IDE, ng/mL, median (QL–QU) | 5.71 (1.92– 7.96) | 3.19 (1.85–6.04) | 0.190 |

In subgroup analyses for cognitive condition, we divided PD patients into two groups based on MMSE test scores and the level of education. We found that serum IDE levels were irrelevant to the impaired cognition. Categorical variables were compared by using the Pearson chi-square test. Continuous variables in normal distribution and homogeneity of variance were compared by using the Student's t-test; otherwise, the Mann-Whitney U test were performed.

**Abbreviation:** PD, Parkinson's disease; AAO, age at onset; LEDDs, levodopa-equivalent daily doses; IDE, insulin-degrading enzyme; MMSE, Mini-Mental State Examination; n, number.

**Table S4.** Serum IDE levels and clinical features according to Hoehn and Yahr stage in patients with PD.

| **Variable** | **PD patients (n = 100)** | | | **HCs (n = 100)** | ***p*** |
| --- | --- | --- | --- | --- | --- |
|  | **Mild (n = 62)** | **Moderate (n = 33)** | **Severe (n = 5)** |  |  |
| Age, y, mean ± SD | 58.23 ± 9.08 | 60.06 ± 9.04 | 65.20 ± 3.70 | 57.44 ± 7.36 | 0.120 |
| AAO, y, mean ± SD | 54.61 ± 9.27 | 54.64 ± 9.20 | 57.40 ± 4.83 | NA | 0.886 |
| Duration of PD, y, mean ± SD (range) | 3.66 ± 2.56 (1–13) | 5.39 ± 3.45 (1–17) | 7.80 ± 4.71 (2–15) | NA | 0.004** |
| MDS-UPDRS I, mean ± SD (range) | 7.32 ± 6.10 (0–26) | 8.88 ± 4.00 (1–19) | 14.20 ± 7.46 (9–27) | NA | 0.005** |
| MDS-UPDRS II, mean ± SD (range) | 11.00 ± 5.42 (1–28) | 15.79 ± 5.79 (7–31) | 35.40 ± 6.47 (26–42) | NA | < 0.001*** |
| MDS-UPDRS III, mean ± SD (range) | 31.85 ± 14.05 (7–65) | 45.85 ± 12.67 (20–78) | 88.60 ± 23.30 (60–116) | NA | < 0.001*** |
| LEDDs, mg, mean ± SD (range) | 315.48 ± 268.20  (0–1250) | 480.61 ± 433.68  (0–2060) | 550.00 ± 274.43  (200–925) | NA | 0.067 |
| MMSE, mean ± SD (range) | 25.06 ± 4.41 (14–30) | 23.24 ± 7.00 (6–29) | 21.80 ± 5.85 (13–28) | NA | 0.257 |
| IDE, ng/mL, median (QL–QU) | 3.39 (1.83– 6.73) | 4.21 (2.06– 6.22) | 5.01 (2.38– 6.05) | 2.76 (1.21–4.89) | 0.844 |

The severity of the disease was assessed by the modified Hoehn and Yahr stage when patients were “off state”. PD patients with Hoehn and Yahr stage 1.0–2.0 were recognized as mild groups. Hoehn and Yahr stage 2.5–3.0 were divided into moderate groups, and Hoehn and Yahr stage 4.0–5.0 were severe groups. Then we demonstrated that IDE was irrelevant to the Hoehn and Yahr stage. Multi-group continuous variables in normal distribution and homogeneity of variance were compared by using the ANOVA; otherwise, the Kruskal-Wallis test were performed. ***P < 0.01, ***P < 0. 001, Kruskal-Wallis test.*

**Abbreviation:** PD, Parkinson's disease; HCs, healthy controls; AAO, age at onset; MDS-UPDRS, Movement Disorder Society-Unified Parkinson’s Disease Rating Scale; LEDDs, levodopa-equivalent daily doses; MMSE, Mini-Mental State Examination; IDE, insulin-degrading enzyme; NA, not available; n, number.

**Table S5.** Clinical features according to Hoehn and Yahr stage in patients with PD.

| **Variable** | ***P* value** | | | | | |
| --- | --- | --- | --- | --- | --- | --- |
|  | **Mild vs**  **Moderate** | **Mild vs**  **Severe** | **Mild vs**  **Control** | **Moderate**  **vs Severe** | **Moderate vs**  **Control** | **Severe vs**  **Control** |
| Duration of PD | 0.024 | 0.051 | NA | 0.788 | NA | NA |
| MDS-UPDRS I | 0.054 | 0.028* | NA | 0.441 | NA | NA |
| MDS-UPDRS II | < 0.001*** | < 0.001* | NA | 0.044* | NA | NA |
| MDS-UPDRS III | < 0.001*** | < 0.001*** | NA | <0.001*** | NA | NA |

The post-hoc Tukey tests or post-hoc Dunn’s test was used for pairwise comparisons after the multiple group comparison for the mild, moderate, and severe groups in PD patients. **P < 0.05, ***P < 0.001, Kruskal-Wallis test and* post-hoc Dunn’s test*.*

**Abbreviation:** PD, Parkinson's disease; MDS-UPDRS, Movement Disorder Society-Unified Parkinson’s Disease Rating Scale; LSD test, post-hoc test (LSD) test; NA, not available; n, number.

**Table S6.** Serum IDE levels and clinical features according to AAO variables in patients with PD.

| **Variable** | **AAO < 55 (n = 42)** | **AAO ≥ 55 (n = 58)** | ***p*** |
| --- | --- | --- | --- |
| Gender, n (female/male) | 17/25 | 30/28 | 0.266 |
| LEDDs, mg, mean ± SD (range) | 438.10 ±404.69(0–2060) | 340.86 ± 280.94 (0–950) | 0.299 |
| Hoehn and Yahr stage, mean ± SD (range) | 1.90 ± 0.66 (1–3) | 2.23 ± 0.95 (1–5) | 0.173 |
| MMSE, mean ± SD (range) | 26.07 ± 4.78 (8–30) | 23.02 ± 5.66 (6–30) | < 0.001*** |
| IDE, ng/mL, median (QL–QU) | 4.01 (1.83– 6.15) | 3.64 (1.90–7.02) | 0.660 |

The AAO-related subgroup was divided according to the median of AAO. We found that IDE were irrelevant to AAO. Categorical variables were compared by using the Pearson chi-square test. Continuous variables in normal distribution and homogeneity of variance were compared by using the Student's t-test; otherwise, the Mann-Whitney U test were performed. ****P < 0.001, Mann-Whitney U test.*

**Abbreviation:** PD, Parkinson's disease; AAO, age at onset; LEDDs, levodopa-equivalent daily doses; MMSE, Mini-Mental State Examination; IDE, insulin-degrading enzyme; n, number.

**Table S7.** Serum IDE levels and clinical features according to duration of PD variables in patients with PD.

| **Variable** | **Duration of PD < 5 (n = 56)** | **Duration of PD ≥ 5 (n = 44)** | ***p*** |
| --- | --- | --- | --- |
| AAO, y, mean ± SD | 56.16 ± 8.15 | 52.98 ± 9.86 | 0.126 |
| LEDDs, mg, mean ± SD (range) | 280.71 ± 252.90 (0–1130) | 510.23 ± 392.61 (0–2060) | 0.001*** |
| Hoehn and Yahr stage, mean ± SD (range) | 1.78 ± 0.72 (1–4) | 2.50 ± 0.85(1–5) | < 0.001*** |
| MMSE, mean ± SD (range) | 24.25 ± 5.48 (6–30) | 24.36 ± 5.58 (7–29) | 0.769 |
| IDE, ng/mL, median (QL–QU) | 4.32 (1.90– 7.42) | 3.36 (1.85–6.09) | 0.594 |

Subgroup analyses uncovered that serum IDE levels were irrelevant to the duration of PD. Continuous variables in normal distribution and homogeneity of variance were compared by using the Student's t-test; otherwise, the Mann-Whitney U test were performed. ****P < 0.001, Mann-Whitney U test.*

**Abbreviation:** PD, Parkinson's disease; AAO, age at onset; LEDDs, levodopa-equivalent daily doses; MMSE, Mini-Mental State Examination; IDE, insulin-degrading enzyme; n, number.

**Table S8.** Serum IDE levels and clinical features according to clinical phenotypes in patients with PD.

| **Variable** | **PD patients (n = 100)** | | | **HCs (n = 100) *p*** | | | | | ***p*** |  |  |
| --- | --- | --- | --- | --- | --- | --- | --- | --- | --- | --- | --- |
|  | **TD (n = 40)** | **IT (n = 17)** | **PIGD (n = 43)** | |  | | |  | |  |  |
| Age, y, mean ± SD | 58.88 ± 7.81 | 59.16 ± 8.89 | 59.94 ± 11.88 | | 57.44 ± 7.36 | 0.787 | | | | |  |
| AAO, y, mean ± SD | 54.70± 8.54 | 54.49 ± 8.72 | 55.59 ± 11.26 | | NA | 0.747 | | | | |  |
| Duration of PD, y, mean ± SD (range) | 4.20 ± 3.14 (1–17) | 4.70 ± 3.30 (1–15) | 4.35 ± 3.04 (1–13) | | NA | 0.759 | | | | |  |
| MDS-UPDRS I, mean ± SD (range) | 6.85 ± 4.34 (0–19) | 9.53 ± 6.40 (1–27) | 7.88 ± 6.34 (1–22) | | NA | 0.158 | | | | |  |
| MDS-UPDRS II, mean ± SD (range) | 12.35 ± 5.56 (1–29) | 15.28 ± 9.66 (2–42) | 13.47 ± 6.51 (3–28) | | NA | 0.719 | | | | |  |
| MDS-UPDRS III, mean ± SD (range) | 37.53 ± 14.77 (8–65) | 42.35 ± 23.15 (7–116) | 35.82 ± 16.97 (9–70) | | NA | 0.649 | | | | |  |
| LEDDs, mg, mean ± SD (range) | 336.38 ± 384.34  (0–2060) | 424.77 ± 298.67  (0–1000) | 379.41 ± 333.02  (0–1130) | | NA | 0.207 | | | | |  |
| Hoehn and Yahr stage, mean ± SD (range) | 1.90 ± 0.68 (1–3) | 2.31 ± 1.02 (1–5) | 2.00 ± 0.41 (1–3) | | NA | 0.260 | | | | |  |
| MMSE, mean ± SD (range) | 24.00 ± 5.94 (6–30) | 24.19 ± 5.72 (7–29) | 25.29 ± 3.70 (15–29) | | NA | 0.980 | | | | |  |
| IDE, ng/mL, median (QL–QU) | 2.93 (1.83– 7.86) | 2.26 (1.71– 5.16) | 4.84 (2.27– 6.23) | | 2.76 (1.21–4.89) | | 0.165 | | | | |

The calculation of Movement Disorder Society-Unified Parkinson’s Disease Rating Scale items (MDS-UPDRS) was used to define tremor dominant (TD), postural instability/gait difficulty (PIGD), and indeterminate (IT) phenotypes of PD patients. Following empirical classification algorithms, the resultant ratio of the mean MDS-UPDRS tremor scores (8 items, 0–4 score for each item) to the mean MDS-UPDRS PIGD scores (5 items, 0–4 score for each item) were developed to define TD patients (ratio ≥ 1.15), PIGD patients (ratio ≤ 0.90), and indeterminate patients (ratios > 0.90 and < 1.15). Specifically, if the numerator of the ratio was zero and the denominator was positive, then these patients were defined as PIGD. Conversely, patients who had a positive numerator with a zero in the denominator were classified. And if both the numerator and denominator of the ratio were zero, the patient was pertinent to the IT phenotype. But we found that discrete clinical phenotypes were irrelevant to IDE. Multi-group continuous variables in normal distribution and homogeneity of variance were compared by using the ANOVA; otherwise, the Kruskal-Wallis test were performed.

**Abbreviation:** PD, Parkinson's disease; HCs, healthy controls; PIGD, postural instability/gait difficulty; IT, indeterminate; TD, tremor dominant; AAO, age at onset; MDS-UPDRS, Movement Disorder Society-Unified Parkinson’s Disease Rating Scale; LEDDs, levodopa-equivalent daily doses; MMSE, Mini-Mental State Examination; IDE, insulin-degrading enzyme; NA, not available; n, number.

**Table S9.** Correlation between serum IDE levels and clinical variables in patients with PD.

| **Variable** | **IDE (ng/ml)** | | |
| --- | --- | --- | --- |
|  | **Rs** | ***P*** | |
| Age | 0.093 | | 0.380 |
| AAO | 0.058 | | 0.581 |
| Duration of PD | 0.007 | | 0.944 |
| MDS-UPDRS I | 0.111 | | 0.291 |
| MDS-UPDRS II | 0.125 | | 0.236 |
| MDS-UPDRS III | 0.178 | | 0.089 |
| LEDDs | 0.175 | | 0.095 |
| Hoehn and Yahr stage | 0.015 | | 0.886 |
| MMSE | -0.230 | | 0.027* |
| FBG | -0.006 | | 0.953 |
| HbA1c | 0.050 | | 0.639 |
| BMI | -0.007 | | 0.948 |
| Weight | -0.105 | | 0.318 |

Binary variables correlation analyses detected the association between serum IDE levels and clinical variables in patients with PD. It revealed that there was a weak but statistically significant negative correlation between serum IDE levels and MMSE scores. ** P < 0.05, Spearman correlation test.*

**Abbreviation:** PD, Parkinson’s disease; IDE, insulin-degrading enzyme; Rs, correlation coefficient; AAO, age at onset; MDS-UPDRS, Movement Disorder Society-Unified Parkinson’s Disease Rating Scale; LEDDs, Levodopa-equivalent daily doses; MMSE, Mini-Mental State Examination; FBG, fasting blood glucose; HbA1c, hemoglobin A1c; BMI, body mass index.
